# Geometric line-of-sight guidance law with exponential switching sliding mode control for marine vehicles’ path following

**DOI:** 10.3389/frobt.2025.1598982

**Published:** 2025-06-23

**Authors:** Chengren Yuan, Changgeng Shuai, Zhanshuo Zhang, Buyun Li, Yuqiang Cheng, Jianguo Ma

**Affiliations:** ^1^ Institute of Noise and Vibration, Naval University of Engineering, Wuhan, China; ^2^ National Key Laboratory on Ship Vibration and Noise, Wuhan, China

**Keywords:** guidance law, line-of-sigh, path following, sliding mode control, marine vehicles, global asymptotic stability

## Abstract

Marine vehicle guidance and control technology serves as the core support for advancing marine development and enabling scientific exploration. Its accuracy, autonomy, and environmental adaptability directly determine a vehicle’s mission effectiveness in complex marine environments. This paper explores path following for marine vehicles in the horizontal plane. To tackle the limitation of a fixed look-ahead distance, we develop a novel geometric line-of-sight guidance law. It adapts to diverse compound paths by dynamically adjusting according to cross-track errors and local path curvature. Then, to enhance control performance, we present an improved exponential switching law for sliding mode control, enabling rapid convergence, disturbance rejection, and chatter reduction. Additionally, integral sliding mode control is integrated to stabilize yaw angular velocity, ensuring the system’s global asymptotic stability. Through a series of numerical simulations, the effectiveness, robustness, and adaptability of our proposed methods are verified.

## 1 Introduction

Marine vehicles, particularly those emphasizing autonomy and intelligence, have gained significant importance (Xu and Pan, 2022; Song et al., 2024a). Unmanned marine vehicles such as USVs and UUVs demonstrate high efficiency in marine resource surveying and development (Wang et al., 2023; Rong and Xu, 2022). However, ensuring optimal control performance remains critical for successful mission execution (Heshmati-Alamdari et al., 2020; Song et al., 2024b).

The control system framework is typically divided into three distinct components: guidance, navigation, and control (GNC) (Fossen, 2011). Uncertainties in models, time-varying oceanic environments, and actuator limitations pose significant challenges for constructing path-following controllers (Kim et al., 2021; Wang et al., 2024). While previous studies, such as Yu et al. (2019); Elmokadem et al. (2017); Lei and Zhang (2017); Reis et al. (2019); Qiao and Zhang (2019), have focused on integrating the guidance and control layers to improve path-following accuracy, many overemphasize control law design for accuracy while neglecting actuator constraints (Yu et al., 2019; Song et al., 2025). Therefore, researching guidance laws to enhance overall GNC system performance and maintain balance among its components is essential.

The line-of-sight (LOS) guidance law is intuitively designed for helmsmen, enabling vehicles to reach desired positions by maintaining alignment with the look-ahead angle (Fossen, 2011; Fossen and Pettersen, 2014). Encarnacao and Pascoal (2000) projected the UUV into a 3D Serrent-Frenet frame and designed a controller integrating the desired path’s kinematic characteristics with the UUV’s dynamic model. However, this method suffered from complexity and singularity issues. To address these, Breivik and Fossen (2005b) introduced a virtual reference point on the desired path within the Serrent-Frenet frame, developing a classical non-singular LOS guidance law for 2D and 3D path following. Yet, this law remained sensitive to ocean currents and used a fixed look-ahead angle. Subsequent studies (Borhaug et al., 2008; Fossen et al., 2014; Fossen and Lekkas, 2017; Miao et al., 2017) focused on mitigating current vulnerability. For instance, Miao et al. (2017) proposed a compound LOS guidance law to estimate sideslip angles and compensate for time-varying current effects in the horizontal plane. Despite these advancements, engineering practice still demands an LOS guidance law with automatic look-ahead angle adjustment. Wang et al. (2022) addressed this by introducing an adaptive LOS guidance law via reinforcement learning, dynamically adjusting the look-ahead angle using a data-driven UUV model. Mu et al. (2018) employed a fuzzy optimization approach to determine optimal look-ahead distances for surface vessels, using Euclidean distances between virtual target points and current positions as fuzzy logic inputs. Xiang et al. (2015) dynamically adjusted look-ahead distances based on path curvature via virtual target points, though these points did not fully reflect real-time vehicle positions.

In the GNC system, control law design is crucial for path following, second only to the guidance system block. Sliding mode control (SMC) is widely adopted to address environmental disturbances and model uncertainties due to its high robustness (Roy et al., 2020). For instance, Elmokadem et al. (2017) proposed terminal SMC (TSMC), fast TSMC (FTSMC), and non-singular TSMC (NTSMC) as effective approaches to reduce following errors under environmental disturbances. To overcome the singularity issue in traditional TSMC, Lei and Zhang (2017) developed an adaptive non-singular integral TSMC scheme, ensuring local finite-time convergence of velocity and position errors to zero. Tutsoy and Barkana (2021) introduced a model-free digital adaptive control for under-actuated manipulators, capable of handling delays, saturations, and uncertainties. This method also extended to chaos control, enabling the learning of unbiased smooth policies in chaotic regions, and real-time experiments verified its accurate long-term prediction and control performance. Moreover, Ma et al. (2023) presented a novel actor-model-critic architecture that combines a neural network model with the traditional actor-critic framework. The neural network model was designed to learn the state transition function, exploring the spatio-temporal variation patterns of the AUV and its surrounding environment.

This paper proposes a novel geometric LOS (GLOS) guidance law and exponential switching law for the horizontal-plane GNC system of unmanned marine vehicles. The objective is to reduce the control layer’s workload and balance the operational burden between the guidance and control laws, thereby enhancing the GNC system’s robustness and adaptability. The desired trajectory is realized by updating the velocity of a virtual target point via the GLOS guidance law, integrated with integral sliding mode control (ISMC) that employs an adaptively adjusted improved exponential switching law. The main contributions are summarized as follows:

• The GLOS guidance law is designed to adjust the look-ahead distance based on both cross-track errors and local path curvature, thereby avoiding the influence of individual factors such as distance (Liu et al., 2017) or curvature (Xiang et al., 2015) alone.
• An enhanced exponential switching law is proposed for general SMC methods. Compared with the conventional exponential switching law, the proposed law demonstrates better performance in rapid convergence, disturbance rejection, and chatter suppression. Based on this, an ISMC law is developed to stabilize the virtual angular velocity of yaw.

The remainder of this paper is organized as follows: Section 2 introduces the notation for path following and the modeling of marine vehicles. The proposed methods are detailed in Section 3. Section 4 then presents the results of numerical simulations, and Section 5 concludes with a comprehensive summary.

## 2 Notation and modeling

### 2.1 Notation

**FIGURE 1:**
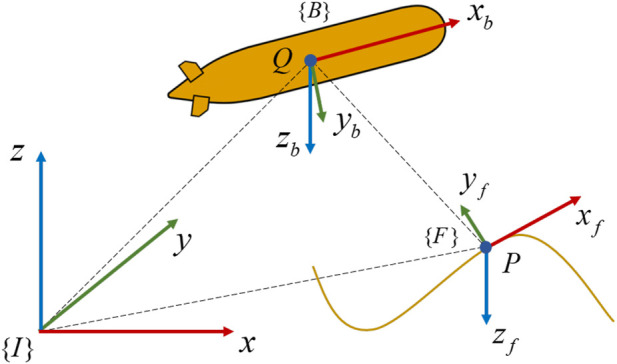
The reference frames for a vehicle.

To construct the coordinate system for path following, the following reference frames, including inertial frame I , body-fixed frame B , Serret-Frenet frame F , and resultant velocity frame V (Encarnacao and Pascoal, 2000), are introduced, as shown in Figure 1. The origin of frame B is set to coincide with the vehicle’s center of buoyancy at $Q={\left[x,y\right]}^{⊤}$ . In the horizontal plane, the 3 degrees of freedom (DOF) kinematic and dynamic models for the under-actuated marine vehicle are configured as (Fossen, 2011).

$$\left\{\begin{array}{c} \dot{x}=u\cos\psi -v\sin\psi \;\\ \dot{y}=u\sin\psi +v\cos\psi \;\\ \dot{\psi }=r\; \end{array}\right.$$ (1)

$$\left\{\begin{array}{c} \dot{u}=f_{u}+g_{u}\tau_{u}+d_{u}\;\\ \dot{v}=f_{v}+d_{v}\;\\ \dot{r}=f_{r}+g_{r}\tau_{r}+d_{r}\; \end{array}\right.$$ (2)

In Equations 1, 2, set $\eta ={\left[x,y,\psi \right]}^{⊤}$ as the vehicle pose in frame {I} , $\nu ={\left[u,v,r\right]}^{⊤}$ as the vehicle velocity in frame {B} , where the azimuth angle is χ=ψ+β , β=arctan(v/u) (Xia et al., 2022). The vector of resultant velocity is defiend as $U_{V}={\left[U,0\right]}^{⊤}$ in frame {V} , where $U=\sqrt{u^{2}+v^{2}}$ . Additionally, $g={\left[g_{u},0,g_{r}\right]}^{⊤}$ , $f={\left[f_{u},f_{v},f_{r}\right]}^{⊤}$ , $d={\left[d_{u},d_{v},d_{r}\right]}^{⊤}$ , and $\tau ={\left[\tau_{u},0,\tau_{r}\right]}^{⊤}$ represent the reciprocal of added mass matrix, corresponding hydrodynamic damping, compound disturbance, and control force and moment (Yuan et al., 2023). Subject to time-varying disturbances, including, uncertain interferences and parameter perturbations, it is denoted that $\dot{d}\neq 0$ .

### 2.2 Control objective

In path following, the marine vehicle with length L aims to follow a predefined path continuously parameterized by a time-independent variable ϖ∈R as $\eta_{d}={\left[x_{d}\left(\varpi \right),y_{d}\left(\varpi \right),\chi_{d}\left(\varpi \right)\right]}^{⊤}$ via a virtual target point $P={\left[x_{d}\left(\varpi \right),y_{d}\left(\varpi \right)\right]}^{⊤}$ , and the time derivative of position vector is ${\dot{P}}_{d}={\left[{\dot{x}}_{d}\left(\varpi \right)\dot{\varpi },{\dot{y}}_{d}\left(\varpi \right)\dot{\varpi }\right]}^{⊤}$ , where ${\dot{x}}_{d}\left(\varpi \right)=\partial x_{d}/\partial \varpi$ , ${\dot{y}}_{d}\left(\varpi \right)=\partial y_{d}/\partial \varpi$ (Xiang et al., 2017). Thus, the referenced azimuth angle is defined as $\chi_{d}=\text{arctan}\left({\dot{y}}_{d}\left(\varpi \right)/{\dot{x}}_{d}\left(\varpi \right)\right)$ . The curvature of referenced path $\kappa_{p}$ should be limited for the inherent constraints of the vehicle as $\kappa_{p}\leq 1/\xi_{\min}$ , where $\xi_{\min}$ is the minimum turning radius of the vehicle. Therefore, the problem of path following has converted that the following error $\eta_{e}=\eta -\eta_{d}$ globally converge to a certain neighborhood of zero within a limited time under bounded disturbances.

## 3 Proposed approach

### 3.1 LOS guidance law and problem

**FIGURE 2:**
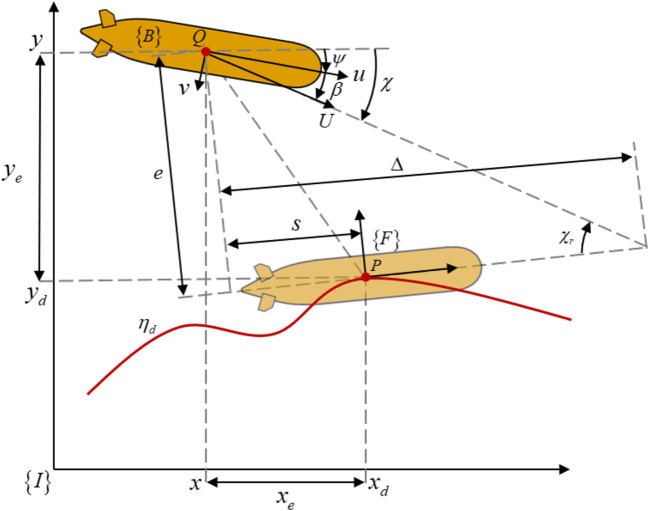
The LOS guidance law for vehicle’s path following control.

As stated in reference Breivik and Fossen (2005b), the controlled vehicle aligns with the look-ahead angle $\chi_{r}$ in the horizontal plane to reach the desired path. As shown in Figure 2, set s and e represent along-track error and cross-track error, the position errors of path following are $P_{e}={\left[s,e\right]}^{⊤}={\left(R_{F}^{I}\right)}^{⊤}\left(Q-P\right)$ , where $x_{e}=x-x_{d}$ and $y_{e}=y-y_{d}$ . According to Yuan et al. (2023), set $R_{V}^{F}={\left[\cos\chi_{r},-\sin\chi_{r};\sin\chi_{r},\cos\chi_{r}\right]}^{⊤}$ as the rotation matrix of frame {V} with respect to frame {F} , and $R_{F}^{I}={\left[\cos\chi_{d},-\sin\chi_{d};\sin\chi_{d},\cos\chi_{d}\right]}^{⊤}$ as the rotation matrix of frame {F} with respect to frame {I} . The time derivative of $P_{e}$ are derived in Equation 3 (Yu et al., 2020).

$${\dot{P}}_{e}=S_{F}^{⊤}P_{e}+R_{V}^{F}U_{V}-U_{d}$$ (3)

where $U_{d}={\left[U_{d},0\right]}^{⊤}$ is the velocity of virtual target point, $U_{d}=\dot{\varpi }\sqrt{{\dot{x}}_{d}^{2}\left(\varpi \right)+{\dot{y}}_{d}^{2}\left(\varpi \right)}$ , $S_{F}={\left[0,-{\dot{\chi }}_{d};{\dot{\chi }}_{d},0\right]}^{⊤}$ is skew-symmetric matrix (Breivik and Fossen, 2005a). To stabilize the position errors of the vehicle, the following Lyapunov function candidate is selected as

$$V_{11}=\frac{1}{2}{\left\|P_{e}\right\|}^{2}$$ (4)

Take the time derivative of Equation 4 and simplify it as

$${\dot{V}}_{11}=s\left(U_{d}\cos\chi_{r}-U_{p}\right)+eU\sin\chi_{r}$$ (5)

To ensure the Lyapunov function Equation 5 is negative-definite, the update rate of P is set as

$$\dot{\varpi }=\frac{U_{d}\cos\chi_{r}+k_{s}s}{\sqrt{{\dot{x}}_{d}^{2}\left(\varpi \right)+{\dot{y}}_{d}^{2}\left(\varpi \right)}}$$ (6)

with the LOS guidance law is designed as

$$\chi_{r}=\arctan\left(\frac{e}{\Delta }\right)$$ (7)

where $k_{s}>0$ , the look-ahead distance is usually set as Δ∈[2L,5L] (Han et al., 2018). Substitute the update velocity Equation 6 and guidance law Equation 7 into Equation 5 as

$${\dot{V}}_{11}=-k_{s}s^{2}-\frac{U}{\sqrt{e^{2}+\Delta^{2}}}e^{2}$$ (8)

In Equation 8, the current LOS guidance law features an indeterminate parameter Δ , which limits its applicability across diverse missions and vehicles. For instance, a larger Δ prolongs adjustment time, while a smaller Δ increases oscillations and overshoots. In the basic LOS guidance law, Δ is typically set as a constant.

### 3.2 GLOS guidance law

**FIGURE 3:**
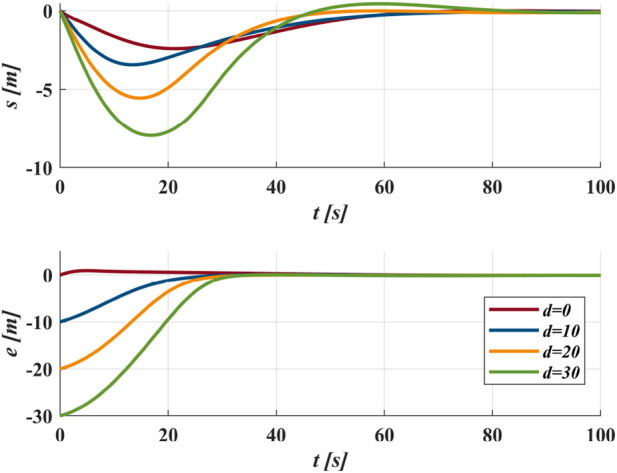
Trace the circular path as $x_{d}^{2}+y_{d}^{2}=900$ with Δ=4L from the different initial positions.

**FIGURE 4:**
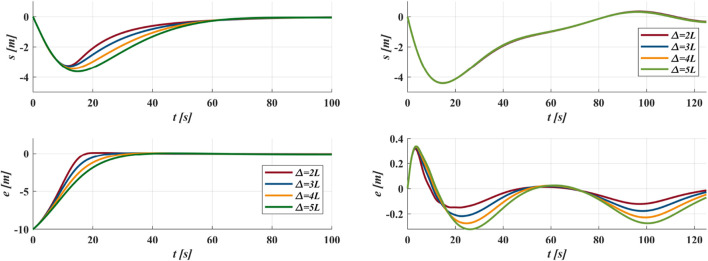
**(a)** Trace the circular path as $x_{d}^{2}+y_{d}^{2}=900$ from initial s=0 and e=10 with different Δ ; **(b)** Trace the sinusoidal path as $y_{d}=10\sin0.05x_{d}$ from initial s=0 and e=0 with different Δ .

In this paper, we explore the geometric relationship between the desired path and vehicle, and further find that the cross-track error e and the local path curvature have more effects on Δ . As shown in Figure 3, when Δ=4L , the vehicle’s steady-state velocity for a circular path is consistent across different horizontal initial positions, though larger e increases overshoot risk. Figure 4 demonstrates that convergence rate decreases with increasing Δ from a fixed position. Also, it illustrates effect of curvature on errors, revealing Δ does not affect along-track error s . Thus, unlike reference Liu et al. (2017), we disregard the influence of s influence on Δ .

**FIGURE 5:**
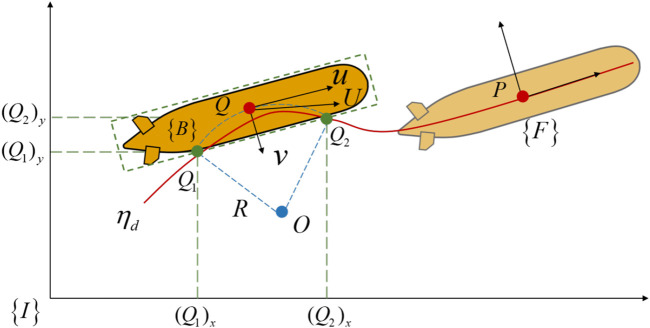
The GLOS guidance law in path following. It use the current path curvature of the vehicle (left vehicle) rather than the virtual target point (right vehicle).

**FIGURE 6:**
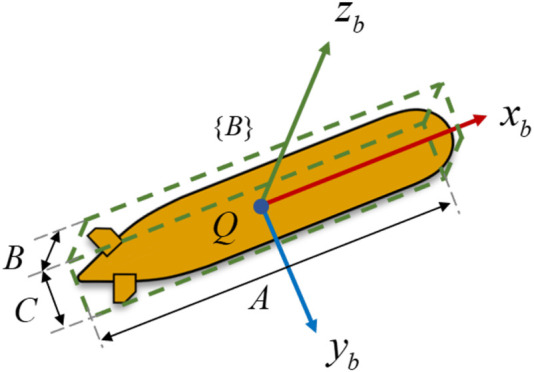
The vehicle in bounding box with parameters.

As for the local path curvature κ , the oriented bounding box (OBB) method is used to efficient calculate the point of intersection around the vehicle in green box, as shown in Figure 5. Set OBB(Q,[ $\overset{⇀}{x_{b}}$ , $\overset{⇀}{y_{b}}$ , $\overset{⇀}{z_{b}}$ ],[A,B,C]) is the function to compute the set of points at the boundary of the bounding box (Ding et al., 2004), where [ $\overset{⇀}{x_{b}}$ , $\overset{⇀}{y_{b}}$ , $\overset{⇀}{z_{b}}$ ] are the unit vectors for three axes in frame {B} , and [A,B,C] are the length, width and height of the bounding box, as shown in Figure 6.

Combining the cross-track error e and the local path curvature κ into LOS guidance law, a novel GLOS guidance law is proposed to adaptively adjust Δ , and the correction of Δ is designed as

$$\Delta =\left\{\begin{array}{cc} \Delta_{\min}+\left(\Delta_{\max}-\Delta_{\min}\right)e^{-\lambda_{1}e^{2}},\; & n_{q}=0\\ \Delta_{\min}+\left(\Delta_{\max}-\Delta_{\min}\right)e^{-\left(\lambda_{1}e^{2}+\lambda_{2}\kappa \right)},\; & n_{q}\geq 1 \end{array}\right.$$ (9)

In Equation 9, $\Delta_{\min}=2L$ , $\Delta_{\max}=5L$ (Han et al., 2018), and $n_{q}$ is the number of intersections between OBB(Q,[ $\overset{⇀}{x_{b}}$ , $\overset{⇀}{y_{b}}$ , $\overset{⇀}{z_{b}}$ ],[A,B,C]) and the desired path. $\lambda_{1}$ and $\lambda_{2}$ are adjustable parameters of e and κ , and the selection of $\lambda_{2}$ often needs to consider the length of vehicle L and $\xi_{\min}$ .

In the GLOS guidance law, κ differs from the reference Xiang et al. (2015). The latter considers the curvature of the virtual target point on a continuous known desired path, as plotted in Figure 5 as P . By comparison, κ in this paper offers the following advantages:

•The method avoids calculating non-existent curvature at the junction of compound paths.
•The approach prevents oscillations at points where curvature abruptly changes in a compound desired path.
•The adjustable parameters B and C enable the vehicle to follow the desired path flexibly.

**FIGURE 7:**
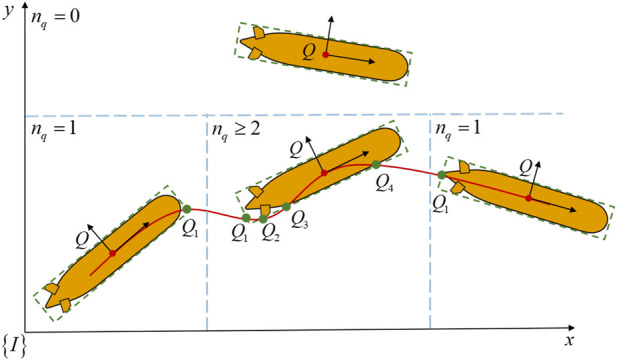
The process of the vehicle following the desired path, it is classified as: (1) Far from the desired path, $n_{q}=0$ ; (2) Enter into the desired path, $n_{q}=1$ ; (3) Following the desired path, $n_{q}\geq 2$ ; (4) Exit out the desired path, $n_{q}=1$ .

Set the number of intersections $n_{q}$ for the desired path and bounding box as $Q=\left[Q_{1},Q_{2},\ldots ,Q_{n}\right]$ . According to $n_{q}$ , it is divided into $n_{q}=0$ , $n_{q}=1$ , and $n_{q}\geq 2$ , as shown in Figure 7. As for $n_{q}=0$ , the vehicle is far from the desired path, and the update of Δ depends on e . When the vehicle approaches the desired path, the desired path and bounding box intersect. The curvature of discrete points is then calculated to correct Δ . As for $n_{q}\geq 2$ , the intersections are sequenced according x value in frame {I} . Set $\kappa =\rho \left(Q_{1},Q_{n}\right)$ is calculated according to the curvature of discrete point $Q_{1}$ , Q , and $Q_{n}$ . If $n_{q}=2$ , κ is calculated with $Q_{1}$ , Q , and $Q_{2}$ , as shown in Figure 5 with blue line. Set $Q_{1}$ and $Q_{2}$ are the intersections, and ${\left(Q_{1}\right)}_{x}<{\left(Q_{2}\right)}_{x}$ . R and O are the radius and center of circumcircle. Theoretically, the desired path and bounding box have infinite intersection points, but due to the curvature constraint in $\kappa_{p}\leq 1/\xi_{\min}$ , there are no more than four intersection points, as shown in Figure 7. Therefore, the calculation of κ is efficient. Especially, as for $n_{q}=1$ , that means the vehicle is approaching to enter or exit the desired path, set κ=0 . Above all, $\kappa \in \left[0,1/\xi_{\min}\right]$ , and the pseudo-code of GLOS guidance law is present in Algorithm 1.

Algorithm 1 GLOS.

**Input:**
   Navigation information $\left[\eta ,\nu \right]={\left[x,y,\psi ,u,v,r\right]}^{⊤}$

   Desired path $\eta_{d}={\left[x_{d},y_{d},\chi_{d}\right]}^{⊤}$

**Output:**
   Look-ahead angle $\chi_{r}$

   Update law $\dot{\varpi }$

1 **Initialize all Parameters;**
2 Calculate $U_{d}$ , s and e ;
3 Update *OBB*(*Q*,[ $\overset{⇀}{x_{b}}$ , $\overset{⇀}{y_{b}}$ , $\overset{⇀}{z_{b}}$ ][*A*,*B*,*C*]);
4 $n_{q}=\eta_{d}\cap OBB$ ;
5 **if** $n_{q}=0$ **then**
6 $\Delta =\Delta_{\min}+\left(\Delta_{\max}-\Delta_{\min}\right)e^{-\lambda_{1}e^{2}}$ ;
7 **else**
8  **if** $n_{q}=1$ **then**
9 κ=0 ;
10  **else**
11 $\kappa =\rho \left(Q_{1},Q_{n}\right)$ ;
12 $\Delta =\Delta_{\min}+\left(\Delta_{\max}-\Delta_{\min}\right)e^{-\left(\lambda_{1}e^{2}+\lambda_{2}\kappa \right)}$ ;
13 $\chi_{r}=\arctan\left(\frac{e}{\Delta }\right)$ ;
14 $\dot{\varpi }=\left(U_{d}\cos\chi_{r}+k_{s}s\right)/\sqrt{{\dot{x}}_{d}^{2}\left(\varpi \right)+{\dot{y}}_{d}^{2}\left(\varpi \right)}$ ;
15 **Repeat**

### 3.3 Controller design

In Section 3.2, only position error is controlled. According to Section 2.2, the heading error $\chi_{e}=\chi -\chi_{d}$ must also be considered. Define the Lyapunov function candidate as $V_{12}=\left(1-\cos\chi_{e}\right)$ . By combining with Equation 4, the Lyapunov function candidate for position and posture is constructed as

$$V_{1}=V_{11}+V_{12}$$ (10)

The time derivative of Equation 10 are derived as

$${\dot{V}}_{1}=-k_{s}s^{2}-\frac{U}{\sqrt{e^{2}+\Delta^{2}}}e^{2}+\left(r-\dot{\beta }-{\dot{\chi }}_{d}\right)\sin\chi_{e}$$ (11)

In order to convert $\chi_{e}$ into the error of virtual angular velocity $r_{e}=r-r_{d}$ according to Equation 11, the referenced virtual angular velocity of yaw $r_{d}$ is designed as

$$r_{d}=\dot{\beta }+{\dot{\chi }}_{d}-k_{r}\sin\chi_{e}$$ (12)

where the control gain is $k_{r}>0$ . Substitute the control law Equation 12 in Equation 11 yields Equation 13 as

$${\dot{V}}_{1}=-k_{s}s^{2}-\frac{U}{\sqrt{e^{2}+\Delta^{2}}}e^{2}-k_{r}\sin^{2}\chi_{e}$$ (13)

In order to stabilize $r_{e}$ , we introduce an ISMC with a novel switching control law to help the sliding mode surface $S_{r}$ related to $r_{e}$ get to zero. The integral sliding surface $S_{r}$ is defined as

$$S_{r}=r_{e}+a_{r}\int_{0}^{t}r_{e}dt$$ (14)

where $a_{r}>0$ is constant. Substitute the dynamic model Equation 2 in the time derivative of Equation 14 yields Equation 15 as

$${\dot{S}}_{r}=f_{r}+b_{r}\tau_{r}+d_{r}-{\dot{r}}_{d}+a_{r}r_{e}$$ (15)

Therefore, the yaw DOF controller is designed as

$$\tau_{r}=\frac{1}{b_{r}}\left({\dot{r}}_{d}-{\hat{f}}_{r}-{\hat{d}}_{r}-a_{r}r_{e}\right)-\frac{1}{b_{r}}f\left(r_{e},S\right)sat\left(r_{e}\right)$$ (16)

$$f\left(r_{e},S_{r}\right)=\frac{\mu }{\sigma +\left(1+{\left|r_{e}\right|}^{-m}-\sigma \right)e^{-n\left|S_{r}\right|}}$$ (17)

In Equations 16, 17, ${\hat{f}}_{r}$ and ${\hat{d}}_{r}$ are the estimation values, and sat() is saturation function according to the reference Patre et al. (2018). μ>0 is coefficient of variational velocity, m>0 and n≥1 are the coefficients of approach. When $r_{e}$ is far away from $S_{r}$ , $r_{e}$ and $S_{r}$ are bigger, that is $\lim_{r_{e},S_{r}\to \infty }f\left(r_{e},S_{r}\right)=\mu /\sigma$ , $r_{e}$ quickly approaches $S_{r}$ . As $r_{e}$ approaches $S_{r}$ , $S_{r}\to 0$ , that is $\lim_{S_{r}\to 0}f\left(r_{e},S_{r}\right)=\mu /\left(1+{\left|r_{e}\right|}^{-m}\right)$ , $r_{e}$ quickly get to original point with suppressing for the chattering problem. To verify the stability of whole system in the horizontal plane, consider the following Lyapunov function candidate as

$$V_{2}=V_{1}+\frac{1}{2}S_{r}^{2}+\frac{1}{2}\varepsilon_{f}^{-1}{\tilde{f}}_{r}^{2}+\frac{1}{2}\varepsilon_{d}^{-1}{\tilde{d}}_{r}^{2}$$ (18)

where the estimation error of $d_{r}$ and $f_{r}$ are ${\tilde{d}}_{r}=d_{r}-{\hat{d}}_{r}$ and ${\tilde{f}}_{r}=f_{r}-{\hat{f}}_{r}$ , the time derivative of Equation 18 is drived and simplified as

$${\dot{V}}_{2}={\dot{V}}_{1}-f\left(r_{e},S_{r}\right)\left|S_{r}\right|+\varepsilon_{f}^{-1}{\tilde{f}}_{r}{\dot{f}}_{r}+\varepsilon_{d}^{-1}{\tilde{d}}_{r}{\dot{d}}_{r}+\varepsilon_{f}^{-1}{\tilde{f}}_{r}\left(\varepsilon_{f}S_{r}-{\dot{\hat{f}}}_{r}\right)+\varepsilon_{d}^{-1}{\tilde{d}}_{r}\left(\varepsilon_{d}S_{r}-{\dot{\hat{d}}}_{r}\right)$$ (19)

In order to set $V_{2}$ negative semi-definite, the adaptive interference laws are designed as

$$\left\{\begin{array}{c} {\dot{\hat{f}}}_{r}=\varepsilon_{f}S_{r}\;\\ {\dot{\hat{d}}}_{r}=\varepsilon_{d}S_{r}\; \end{array}\right.$$ (20)

Substitute the adaptive interference law Equation 20 in Equation 19 as

$${\dot{V}}_{2}\leq -k_{s}s^{2}-\frac{U}{\sqrt{e^{2}+\Delta^{2}}}e^{2}-k_{r}\sin^{2}\chi_{e}-f\left(r_{e},S_{r}\right)\left|S_{r}\right|+\varepsilon_{f}^{-1}{\tilde{f}}_{r}{\dot{f}}_{r}+\varepsilon_{d}^{-1}{\tilde{d}}_{r}{\dot{d}}_{r}$$ (21)

In Equation 21, according to the reference Yuan et al. (2022), ${\tilde{d}}_{r}{\dot{d}}_{r}\leq 0$ and ${\tilde{f}}_{r}{\dot{f}}_{r}\leq 0$ . Also, $f\left(r_{e},S_{r}\right)>0$ , and ${\dot{V}}_{2}\leq 0$ . If and only if s=0 , e=0 , $\chi_{e}=0$ , and $S_{r}=0$ , that ${\dot{V}}_{2}=0$ . The control system converges asymptotically according to the Lyapunov stability theorem. For the surge velocity u , the PID controller or the dynamic controller in Equation 16 stabilize the error of surge velocity $u_{e}$ . Therefore, this paper will not do too much elaboration.

## 4 Numerical simulations

To verify the GLOS guidance law and improved exponent switching law for path following in the horizontal plane, this paper takes REMUS 100 AUV as the research object and adopts the hydrodynamic parameters from reference Prestero (2001). The main parameters of the proposed scheme are as follows: L=1.33 , $k_{s}=0.1$ , $k_{r}=2$ , $\lambda_{1}=5$ , $\lambda_{2}=30$ , $a_{r}=5$ , μ=1 , m=1 , n=2 , σ=1 , $\varepsilon_{f}=7$ , and $\varepsilon_{d}=3$ . All the simulation were impacted by unknown interferences as $d_{u}=0.2\sin\left(0.05t+\pi /3\right)+n\left(t\right)$ , $d_{v}=0.1\sin\left(0.04t+\pi /3\right)+n\left(t\right)$ , and $d_{r}=0.2\sin\left(0.05t+\pi /3\right)+n\left(t\right)$ , where E[n(t)]=0 . All the initial velocity and angular velocity were set as ${\left[u,v,r\right]}^{⊤}={\left[0,0,0\right]}^{⊤}$ .

### 4.1 Case I

Case I employs a compound straight-line and curve desired path to verify the effectiveness of the proposed method. Four different methods track the compound desired path starting from ${\left[x,y,\psi \right]}^{⊤}={\left[5,-5,\pi /2\right]}^{⊤}$ : (1) The proposed method, which is the GLOS guidance law with an improved exponent switching law based on the SMC; (2) Method 1, the LOS guidance law with a traditional switching law based on the SMC; (3) Method 2, the LOS guidance law with an improved exponent switching law based on the SMC and a fixed Δ=2L ; (4) Method 3, the LOS guidance law with an improved exponent switching law based on the SMC and a fixed Δ=5L . The desired path is set as Equation 21

$$y_{d}=\left\{\begin{array}{cc} \frac{4}{3}x_{d},\; & 0\leq x_{d}<30\\ {\left(1600-{\left(x_{d}-70\right)}^{2}\right)}^{\frac{1}{2}}+40,\; & 30\leq x_{d}\leq 110\\ \frac{4}{3}\left(100-x_{d}\right)+40,\; & 110<x_{d}\leq 140 \end{array}\right.$$ (22)

**FIGURE 8:**
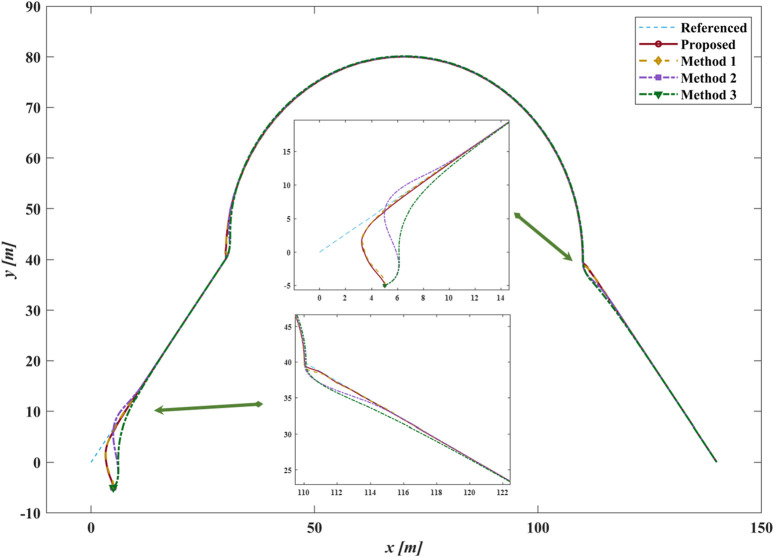
The path following results of different methods in case I.

**FIGURE 9:**
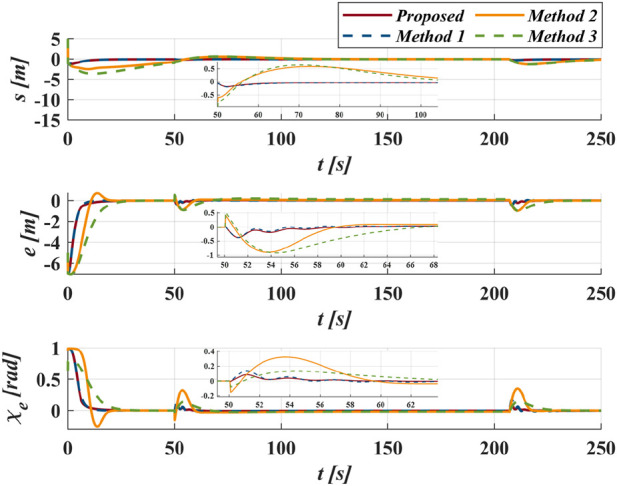
The following errors of different methods in case I.

**FIGURE 10:**
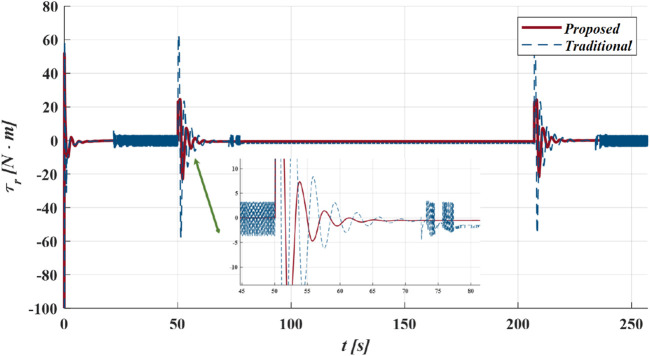
The control moments of yaw $\tau_{r}$ for proposed method and method 1 in case I.

As shown in Figure 8, for Methods 2 and Methods 3, a smaller Δ leads to slower convergence to the desired path, while a larger Δ causes overshoots in the initial phase under the same control law. In contrast, the proposed method and Method one utilize the GLOS guidance law, which helps the vehicle avoid the slow convergence and overshoot issues mentioned above, particularly at the initial position and turning points. Figure 9 displays more details about the following errors of different methods. As shown in Figure 10, compared with Method 1, the improved exponent switching law significantly suppresses $\tau_{r}$ oscillations under the same parameters and quickly stabilizes $r_{e}$ with strong anti-disturbance performance.

### 4.2 Case II

Case II uses the sinusoidal desired path, which is $y_{d}=20\sin0.03x_{d}$ , to verify the robustness of proposed method. The desired path is tracked by proposed method from different initial position as: (1) e=0 , ${\left[x,y,\psi \right]}^{⊤}={\left[0,0,\pi /2\right]}^{⊤}$ ; (2) e=5 , ${\left[x,y,\psi \right]}^{⊤}={\left[0,-5,\pi /2\right]}^{⊤}$ ; (3) e=10 , ${\left[x,y,\psi \right]}^{⊤}={\left[5,-10,\pi /2\right]}^{⊤}$ ; (4) e=15 , ${\left[x,y,\psi \right]}^{⊤}={\left[10,-15,\pi /2\right]}^{⊤}$ .

**FIGURE 11:**
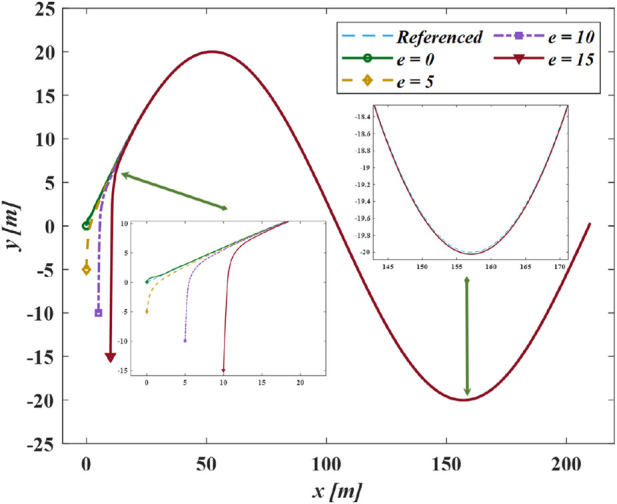
The following results of different position in case II.

**FIGURE 12:**
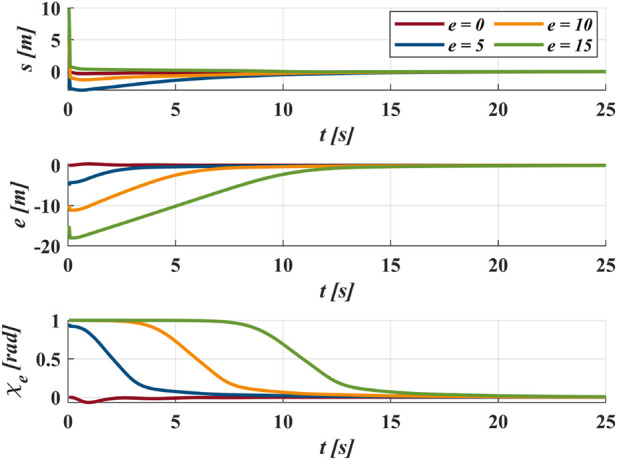
The following errors of different position in case II.

**FIGURE 13:**
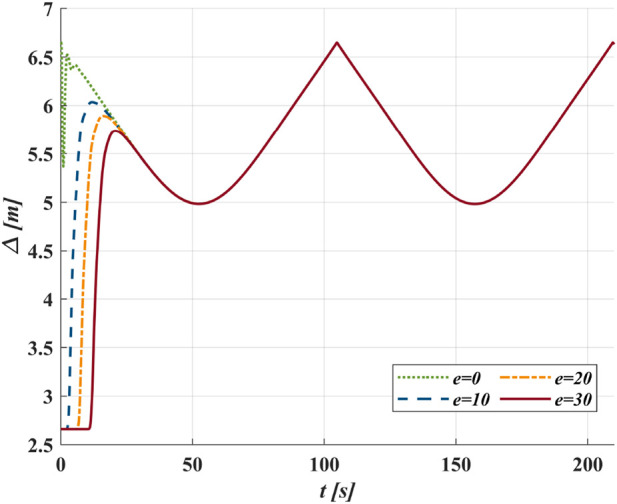
The change of Δ for different cross-track error e in case II.

Figure 11 demonstrates the proposed method applied under different initial positions. The GLOS guidance efficiently directs the vehicle regardless of the initial cross-track error distance e . As shown in Figure 12, even when following a desired path with variable curvature, the following errors stabilize at all positions, particularly at the start position and corners with larger curvature. As shown in Figure 13, at the beginning of the following, the change of Δ is mainly affected by cross-track error e , Δ increase with the decrease of e . After following up the desired path, the change of Δ is mainly affected by κ . Δ decrease with the increase of κ . In general, the change of Δ better helps the vehicle to realize path following control.

## 5 Conclusion

The guidance layer and control layer enhance the GNC system capabilities of marine vehicles. This paper presents a balanced approach, integrating guidance and control law calculations, to boost the GNC system’s robustness and adaptability, instead of merely optimizing the control law. To overcome the fixed look-ahead distance limitation, a novel GLOS guidance law is proposed. It can adaptively adjust according to the cross-track error and the curvature of nearby points, enabling the marine vehicles to handle various compounded paths. This law outperforms traditional LOS guidance laws in several aspects. For the control law, an improved exponential switching law based on the ISMC method stabilizes the yaw’s virtual angular velocity, featuring rapid convergence, anti-disturbance, and chatter suppression. The Lyapunov stability theorem verifies the global asymptotic stability of the designed system. Simulation results confirm the robustness and adaptability of these proposed schemes.

Future work will focus on verifying the proposed methods through actual tests using various vessels (e.g., AUVs and USVs), with validation conducted on these platforms.

## Data Availability

The original contributions presented in the study are included in the article/supplementary material, further inquiries can be directed to the corresponding authors.
